# Design and Application of Conjugated Oligomers for Imaging and Anticancer and Antibacterial Treatment

**DOI:** 10.3390/molecules30183719

**Published:** 2025-09-12

**Authors:** Jianing Wang, Min Ma, Yuxuan Ding, Lili Luo, Yilin Zuo, Libing Liu

**Affiliations:** 1 Department of Nutrition and Health, China Agricultural University, Beijing 100193, China; jianing20010726@126.com (J.W.); mamin_st@163.com (M.M.); dingyxuan2024@163.com (Y.D.); lililuo_w@163.com (L.L.); 18856313363@163.com (Y.Z.); 2 Key Laboratory of Precision Nutrition and Food Quality, China Agricultural University, Beijing 100193, China

**Keywords:** conjugated oligomers, detection, anticancer, antibacterial

## Abstract

The excessive use of antibiotics and the rise in resistant strains have significantly contributed to increased mortality rates associated with tumors and infectious diseases, posing a severe threat to public health. Consequently, there is an urgent need to enhance detection and treatment strategies for critical diseases, including cancer and pathogenic microorganisms. Conjugated oligomers, formed by a limited number of repeating conjugated units linked through covalent bonds, exhibit a high potential for various applications in biological monitoring and disease diagnosis due to their controllable molecular weight, precise molecular structure, and excellent photostability. Therefore, it is particularly essential to design and optimize the structure of conjugated oligomers to improve their biocompatibility and therapeutic efficacy. In this review article, we summarize recent advancements in applying conjugated oligomers in imaging, cancer therapy, and antibacterial treatment. Furthermore, this article critically examines the challenges in their biological applications, underscoring the need for continued innovation.

## 1. Introduction

Malignant tumors and pathogenic microbial infections represent significant global public health challenges. According to research by the World Health Organization, the number of cancer-related deaths in China was 3,002,899 in 2020. It is estimated that by 2040, the number of newly diagnosed cancer patients each year will reach 28.4 million, representing a 47% increase from that of 2020 [1]. In addition, antimicrobial resistance (AMR) has emerged as a critical threat, with 2021 global estimates attributing 4.71 million fatalities to drug-resistant bacterial infections, including 1.14 million deaths directly caused by AMR pathogens [2]. In recent years, various innovative treatment modalities have emerged in treating malignant tumors and pathogenic microbial infections, including photodynamic therapy (PDT) and photothermal therapy (PTT) [3,4,5,6,7,8]. However, the clinical management of neoplastic diseases faces three formidable challenges: (1) the inherent limitations in achieving timely tumor detection during early-stage progression, (2) the elevated propensity for metastatic dissemination and disease recrudescence during therapeutic convalescence, and (3) the alarming emergence of antimicrobial resistance patterns. These persistent clinical hurdles have consequently propelled the development of novel and precision-driven diagnostic–therapeutic paradigms, coupled with the innovation of highly efficacious and clinically applicable diagnostic reagents and therapeutic agents, to the forefront of translational research priorities. Such innovations promise not only to enhance therapeutic outcomes and quality of life for patients but also to establish novel paradigms in clinical practice. The integration of molecular diagnostics with multifunctional therapeutic platforms may ultimately provide a transformative approach to managing complex diseases, representing a crucial frontier in contemporary biomedical research.

Conjugated oligomers (COs), a class of organic semiconductor materials with exact molecular structures, have gained significant attention thanks to their adjustable conjugated lengths and unique electronic properties. They demonstrate broad spectral absorption and high extinction coefficients, excelling in light harvesting and intermolecular energy transfer. When excited, COs primarily undergo radiative decay via fluorescence from the π-conjugated backbone. Non-radiative decay can also occur, either through heat generation or energy/electron transfer to acceptors like dyes or oxygen. These characteristics enhance the signal-to-noise ratio of detection signals, presenting a new strategy for ultrasensitive biosensing [9].

Owing to their distinctive structural features, COs exhibit exceptional capabilities in light absorption and electron transport, which have led to their widespread application in biological imaging, antibacterial treatments, and anticancer therapies. In the field of bioimaging, COs are increasingly employed as alternatives to conventional organic fluorescent dyes. Their high fluorescence quantum yield and superior photostability enable long-term tracking of organelles, cell membranes, and tumor tissues in vivo. Current research is focused on developing COs with absorption wavelengths falling within the second near-infrared window (NIR-II) to facilitate deeper tissue penetration and enhance imaging signal-to-noise ratios. In therapeutic contexts, COs serve as highly efficient photosensitizers that, upon light irradiation, generate reactive oxygen species (ROS) or substantial heat, leading to rapid and broad-spectrum disruption of bacterial cell membranes and biomacromolecules. This multi-target mechanism markedly reduces the likelihood of resistance development. Furthermore, a growing number of multifunctional theranostic platforms based on COs have been constructed, enabling the synergistic integration of PDT, PTT, chemotherapeutic drug delivery, and immune activation within a single system. By combining multiple treatment modalities, such platforms can overcome limitations imposed by the microenvironment, significantly enhance therapeutic outcomes, and suppress disease metastasis—representing a highly promising strategy in the realm of precision medicine [10,11,12].

Notably, the molecular backbones and terminals of COs can be precisely functionalized through modular synthesis. Functional groups such as sulfonic acid groups, quaternary ammonium salts, or targeting ligands can be introduced, improving their water solubility, biocompatibility, and specific recognition abilities. This molecular designability grants COs multidimensional application potentials in the field of biomedicine. They can be used for tumor-targeted PDT, specific recognition and inactivation of pathogenic bacteria, and high-resolution bioimaging. With their precisely controllable structures and strong performance tunability, COs provide an ideal material basis for constructing new integrated diagnosis and treatment platforms, advancing the development of precision medicine toward molecular-level regulation.

In this review, we comprehensively examines the innovative biomedical applications of COs, covering the (1) sensing of pathogenic microorganisms, tumors, and their biomarkers; (2) eradication of pathogenic microbial infections; and (3) effective elimination of tumor cells through tumor microenvironment regulation. Moreover, we delve into the current research landscape, limitations, and future prospects of COs. To facilitate comparative analysis of the structure–activity relationship between material properties and therapeutic efficacy, and to guide the development of novel COs, the review includes a table summarizing the action mechanisms of different COs, along with their fluorescence absorption wavelengths, material properties, application types, and application subjects.

## 2. Conjugated Oligomers

COs are short-chain π-conjugated systems comprising a finite number of conjugated repeating units (typically ranging from several to dozens), which are constructed through precise synthetic routes. In contrast to conjugated polymers-characterized by high molecular weights and broad molecular weight distributions—COs possess well-defined and readily tunable chemical structures. Their molecular size places them between conventional small molecules and polymers, endowing COs with structural precision, excellent batch-to-batch reproducibility, and extended π-electron delocalization [13,14,15].

**Figure 1:**
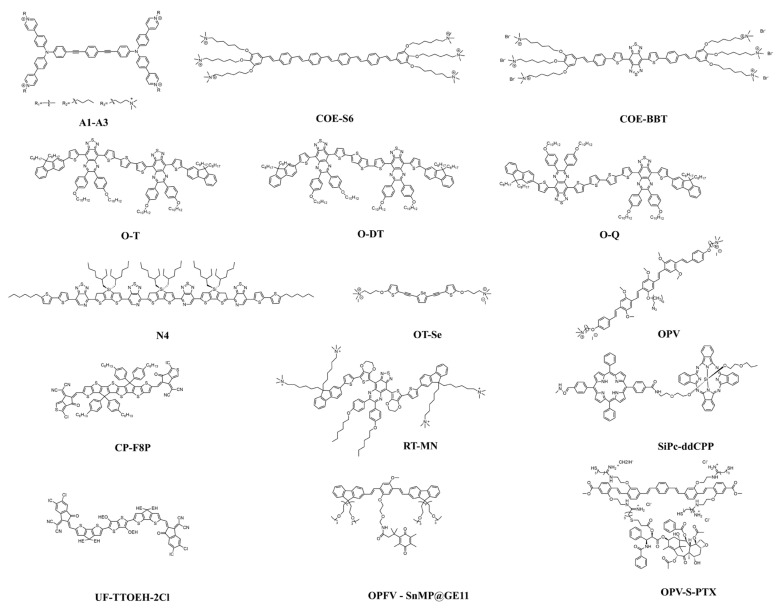
Chemical structure of COs.

From the perspective of structural design and functionalization, COs can be strategically engineered via molecular design to achieve diverse functionalities (Figure 1). For example, oligofluorene-based COs functionalized with sulfonic acid groups exhibit improved water solubility and biocompatibility, rendering them suitable for cellular imaging and biosensing applications. Quaternary ammonium-modified oligothiophene COs demonstrate efficient photodynamic antibacterial activity, enabling simultaneous pathogen recognition and eradication. COs featuring a donor–acceptor–donor (D-A-D) architectural motif facilitate excitation across a narrow optical bandgap and NIR-II emission, thereby offering enhanced infrared absorption and emission properties. Terminal charged side chains confer direct water solubility without the need for organic co-solvents or encapsulation, while promoting electrostatic and hydrophobic interactions with bacterial membranes. This enhances membrane penetration and facilitates bacterial eradication. Furthermore, thiol-functionalized COs exhibit improved aqueous solubility and fluorescence properties, enabling efficient cellular uptake and targeted therapeutic delivery [16,17,18,19,20,21,22].

COs display broad UV–visible absorption profiles and efficient excited-state electron transport. Upon photoexcitation, electrons migrate rapidly along the conjugated backbone toward energy acceptors or reactive sites, leading to significant amplification of optical signals and improved detection sensitivity [15]. In comparison to conjugated polymers, which often exhibit structural heterogeneity, COs offer distinct advantages including precise structural control, tunable energy levels and spectral properties, as well as simplified purification and characterization processes. Relative to inorganic nanomaterials, COs generally demonstrate superior biocompatibility, reduced toxicity, and minimal fluorescence blinking. Nevertheless, challenges remain, such as restricted absorption/emission wavelengths due to limited conjugation lengths, and aggregation-caused quenching in condensed or aggregated states—issues that may be alleviated through rational molecular design [14].

In summary, COs represent a class of functional materials that bridge the gap between small molecules and polymers, offering well-defined structures, outstanding optoelectronic properties, and highly adaptable modification strategies. They demonstrate significant potential for applications in biomedicine and optoelectronics. Continued advances in molecular engineering are expected to further broaden the functional performance and applicability of COs.

## 3. Detection Based on Conjugated Oligomers

### 3.1. Fluorescence Detection

Fluorescent detection stands out among biosensing technologies due to its high sensitivity, selectivity, real-time capability and non-invasiveness [23]. However, traditional small-molecule fluorescent dyes have disadvantages, like suboptimal spectral properties and poor photostability, which restrict their application in detection and real-time imaging [24]. Yu et al. developed a ternary fluorescent sensor array by self-assembling fluorophores and cucurbit [7] uril (CB [7]) for bacterial detection (Table 1). Poly (p-arylethylene) (PPE) is a typical aggregation-caused quenching (ACQ) fluorophore. After forming a complex with aggregation-induced luminescence (AIE), it exhibits obvious quenching fluorescence. However, when complexes with opposite optical properties coexist with bacteria, they exhibit signal enhancement effects. Therefore, the author designed and constructed a novel complex and simultaneously introduced non-specific cationic substituted side chains with different physicochemical properties at the four symmetrical ends to enhance the binding ability to bacteria and the detection sensitivity. Results showed the supramolecular array generated unique bacterial fingerprints, identified mixed bacterial species, and distinguished 10 bacterial strains in aqueous and urine media [25]. Despite high sensitivity, the sensor’s operation is relatively complex. Therefore, developing a simple and effective fluorescent detection method for bacterial classification is crucial. The classic Gram staining method has limitations, such as its significant operational impact and difficulty in allowing for real-time live bacteria observation. Zhou et al. designed the fluorescent membrane probe Conjugated Oligoelectrolyte (COE) COE-S6 to differentiate Gram-positive and Gram-negative bacteria. Gram-negative bacteria have a characteristic asymmetric outer membrane, with lipopolysaccharide (LPS) on the outer leaflet, which can act as a molecular sieve to protect cells from chemical stress. The presence of LPS hinders the insertion of COE-S6 membranes, resulting in a weak fluorescence signal. The two types of Gram bacteria in the mixture can be separated and labeled by re-staining COE-S6 with the non-specific and lipophilic FM 4-64 membrane probe. Meanwhile, COE-S6 does not inhibit bacterial growth and is easy to use, making it a promising membrane-specific fluorescent probe, offering potential for the in situ monitoring of living cell behavior [26].

**Figure 2:**
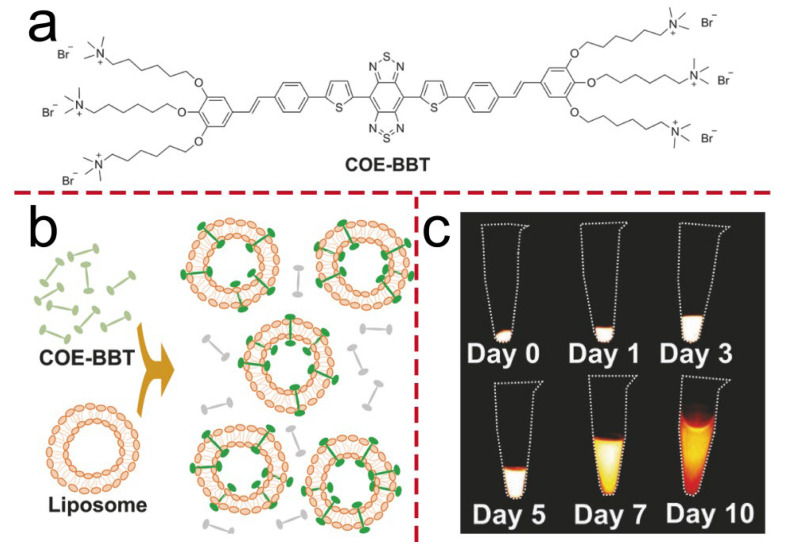
(**a**) The chemical structure of COE-BBT. (**b**) Schematic diagram of the membrane embedding of COE-BBT and the fluorescence “turn-on” based on the model membrane of small unilamellar vesicles. (**c**) Fluorescence images of COE-BBT-stained C6-Luc glioma cells after growth in vitro for different times.

In fluorescence imaging, light in the near-infrared second window (NIR-II) offers superior performance, characterized by deeper tissue penetration and higher imaging clarity [35,36,37]. Zhou et al. synthesized a conjugated oligoelectrolyte COE-BBT, featuring a thiophene-benzothiadiazole-thiophene (donor–acceptor–donor, D-A-D) core fragment (Figure 2a). When COE subclasses are introduced as membrane-inserting molecules, they exhibit low cytotoxicity, particularly when their molecular length is comparable to the thickness of the lipid bilayer. Furthermore, the fluorescence intensity of COE increases in lipophilic environments, making them suitable for use as fluorescent dyes. The structural configuration of COE-BBT contributes to its specificity for lipid bilayers. In aqueous environments, the background fluorescence is minimal, with the compound predominantly retained at the target site, offering advantages in terms of usability. It has two emission peaks at 503 nm and 1020 nm. The high-energy excitation wavelength enables subcellular analysis in vitro using a conventional confocal microscope, while the low-energy excitation wavelength supports in vivo NIR-II imaging. Intracranial (14-day) and subcutaneous (26-day) tumor models (Figure 2c) have shown that, compared with traditional exogenous fluorescent probes, the NIR-II emission intensity of COE-BBT increases with tumor growth. This phenomenon may be attributed to the gradual reduction in the self-quenching effect of COE-BBT within cellular environments. This suggests that membrane-embedded COE holds great potential for long-term in vivo biological process monitoring [20]. Liu et al. developed a novel NIR-II photothermal agent for theranostics. Its core structure is S-D-A-D’-A-D-S, where S is a fluorenyl shielding unit, D is a thiophene donor, A is a thiophenoquinoxaline acceptor, and D’ is a thiophene-based linking donor with variable conjugation lengths. By adjusting D’, three oligomers (O-T, O-DT, O-Q) were synthesized using terthiophene, thieno [3,2-b] thiophene (TT), and quaterthiophene, respectively. The modification of D’ can effectively regulate the molecular configuration and energy levels, thereby influencing its optical and thermal properties. Compared with traditional organic chemical materials, such as polymethyl alkyl small molecules, the D-A-D molecular architecture exhibits superior chemical and optical stability. This structure can efficiently promote electron delocalization between the donor and acceptor units, enhance intramolecular charge transfer, and broaden the light absorption spectrum. Their nanoparticles (NPs) show NIR-II absorption and fluorescence emission at 1064 nm excitation. Among them, the molar extinction coefficient (ε) of O-T NPs at 1064 nm reaches 1.55 × 10^4^ M^−1^·cm^−1^, which is 4.3–4.8 times that of the other two kinds of nanoparticles, and it has the highest fluorescence brightness. Experiments show that O-T NPs enable high-resolution in vivo NIR-II imaging at 1064 nm laser excitation, with a penetration depth of up to 9 mm. This study confirms the feasibility of the molecular design and offers new ideas for CO applications in the biological field [27]. The D-A-D molecular structure can effectively achieve the redshift of chemiluminescence emission and can effectively overcome the problem that the emission wavelengths of most chemiluminescence systems are mainly limited to the visible light region. Tang et al. developed a chemiluminescent probe, termed TBL, by conjugating luminol with benzothiazole and triphenylamine derivatives for the detection and imaging of singlet oxygen (^1^O_2_). Through extension of the conjugated framework of luminol, chemiluminescence emission in the near-infrared-I (NIR-I) region was successfully achieved. Nevertheless, despite employing a molecular conjugation expansion strategy, the emission wavelength of the probe remained outside the NIR-II window. To address this limitation, a CRET-FRET cascade strategy was implemented. Several CRET and FRET donor-acceptor pairs were constructed, leading to the development of a NIR-II chemiluminescent nanosensor, designated OLBB-CLS, based on a sequential CRET-FRET energy transfer mechanism. The chemiluminescent response of OLBB-CLS toward various ROS was systematically evaluated. The nanoparticles exhibited markedly enhanced luminescence in the presence of ^1^O_2_ compared to other ROS, including H_2_O_2_, hydroxyl radicals (·OH), hypochlorite (ClO^−^), and superoxide anions (O_2_·^−^). In contrast to conventional luminescent materials, OLBB-CLS demonstrates high selectivity for ^1^O_2_, showcasing excellent specific chemiluminescent properties. Moreover, this nanosensor exhibits favorable biocompatibility and enables high signal-to-noise ratio NIR-II chemiluminescence imaging in vivo within ^1^O_2_-associated disease models [38].

### 3.2. Photoacoustic Detection

In photoacoustic detection, contrast agents absorb laser light and generate acoustic waves, which are detected and converted into images based on their arrival time. Unlike light, sound waves scatter less in biological tissues, giving photoacoustic detection advantages like high tissue contrast, spatial resolution, and deep imaging capability [39,40]. Cai et al. developed a CO N4 NPs. Compared with traditional near-infrared absorption conjugated polymers (such as polypyrrole and poly (3,4-ethylenedioxythiophene) (PEDOT)), COs have a more definite molecular structure, can be purified to a greater extent, have stronger molecular design potential, and can achieve absorbance in the near-infrared range, as well as having effective photoacoustic (PA) signal generation and good biocompatibility. Using DSPE-PEG2000 as the encapsulation matrix, surface functionalization was achieved via a straightforward precipitation method. The PA signal intensity peaks 10 min N4 NPs injection and remains at 45% of the maximum even after 60 min. The excellent in vivo photostability of N4 NPs enables continuous PA imaging, which is promising for clinical applications [28].

This subsection elaborates on the state-of-the-art applications of COs in fluorescence and photoacoustic imaging. In comparison with conventional materials, COs exhibit pronounced advantages for cellular imaging: their well-defined and highly designable molecular structures enable tailored material design for specific cellular targets, resulting in high selectivity and targeting precision. These materials possess outstanding optical properties, including strong absorption and high photostability in the NIR-II, coupled with deep tissue penetration and high-resolution imaging capabilities. Furthermore, they demonstrate excellent biocompatibility and low toxicity. For photoacoustic imaging, COs exploit their efficient photothermal conversion capability. The detection of resulting ultrasound waves combines the high contrast of optical methods with the deep penetration of acoustic techniques, offering a powerful hybrid modality for biomedical visualization.

## 4. Disease Treatment with Conjugated Oligomers

### 4.1. Antibacterial

COs mediate bacterial eradication through a multi-targeted and rapid approach, leveraging photodynamic action (generating reactive oxygen species), photothermal effects (producing localized hyperthermia), and an inherent physical membrane-disruption mechanism. This non-specific mode of attack substantially reduces the likelihood of inducing bacterial resistance, thereby presenting a promising strategy to combat the global challenge of antibiotic resistance.

#### 4.1.1. Photodynamic Therapy

In recent years, PDT has gained significant attention owing to its advantages such as excellent spatiotemporal precision, low risk of drug resistance development, and broad antibacterial spectrum [41,42,43]. The core mechanism of photodynamic antibacterial action initiates with photoabsorption and electronic transition. Upon irradiation with light of an appropriate wavelength, COs absorb photon energy, promoting electrons from the highest occupied molecular orbital (HOMO) to the lowest unoccupied molecular orbital (LUMO). Subsequently, the triplet-state photosensitizer molecules engage with environmental oxygen via two primary pathways: the Type II process generates increased ^1^O_2_, while the Type I pathway (secondary route) produces other ROS, such as O_2_^−^ and·OH. Ultimately, the abundant ROS elicit indiscriminate oxidative damage across multiple bacterial cellular targets. This includes disruption of cell membranes, protein inactivation, lipid peroxidation, and degradation of genetic material such as DNA. Owing to its multi-target and physico-chemical nature, this oxidative assault poses significant challenges for the development of bacterial resistance. Thus, it constitutes the fundamental rationale behind the high efficacy and reduced propensity for resistance induction observed in photodynamic antibacterial therapy [44,45,46]. Thus, the ROS-generating capacity of photosensitizers within cells is crucial for PDT’s efficacy. COs have excellent photophysical properties and strong ROS generating abilities both in vitro and in vivo, making them promising for antibacterial and antitumor treatments.

**Figure 3:**
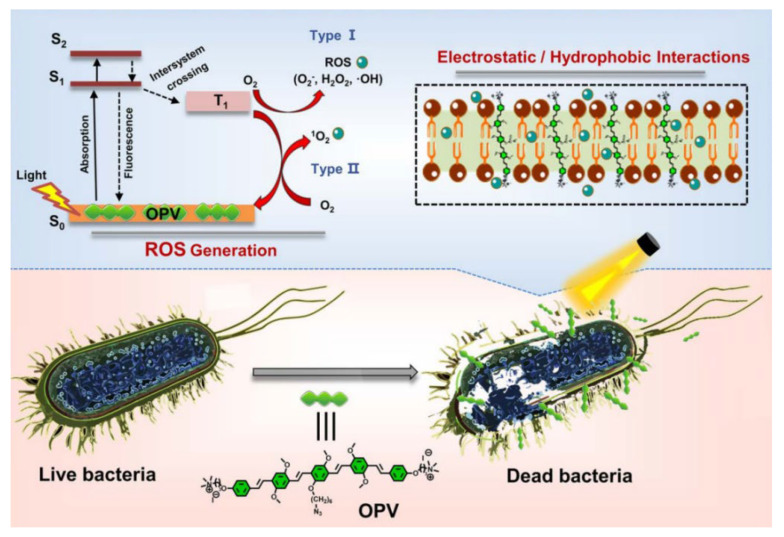
The PDT mechanism of OPV.

Singlet oxygen (^1^O_2_), with its potent oxidative capacity, effectively eradicates bacteria and tumor cells. However, its generation requires sufficient oxygen, which is often limited in the hypoxic microenvironments of tumor tissues or bacterial infection sites, thereby restricting PDT efficacy. In contrast, type I photosensitizers produce free radicals like superoxide and hydroxyl radicals, which are less oxygen-dependent [47,48]. Zhao et al. developed a novel type I photosensitizer, oligo (thienyl ethynylene) modified by selenium (Se) (OT-Se), to address the limitations of traditional photosensitizers in generating ^1^O_2_ within hypoxic infected lesions. The OT-Se molecular design is derived from oligomers (thioenyl acetylene) (OT-S), featuring excellent visible light capture capability and high-performance ^1^O_2_ generation. By introducing selenium atoms, OT-Se enhances the generation efficiency of O_2_^−^ and OH and reduces dependence on the oxygen level in the cellular microenvironment. In addition, cationic quaternary ammonium salts are coupled to both ends of OT-Se to provide reliable bacterial membrane anchoring, demonstrating potent antibacterial activity against multidrug-resistant bacteria such as methicillin-resistant Staphylococcus aureus (MRSA) and carbapenem-resistant Escherichia coli (CREC) at the sub-micromolar level under white light irradiation at 30 mW/cm^2^. Experiments show that OT-Se has excellent water dispersibility, bacterial membrane anchoring ability, and biocompatibility. Through photodynamic action, it disrupts the bacterial membrane structure and achieves complete clearance of drug-resistant bacterial infected wounds in vivo. This study offers a molecular design strategy for efficient type I photodynamic antibacterial agents and new ideas for combating drug-resistant bacterial infections [11]. Wang et al. developed water-soluble oligo phenylene vinylene (OPV) through end-functionalization (Figure 3). The two terminal ends of the CO OPV are functionalized with positively charged quaternary ammonium groups, which significantly enhances its water solubility. These groups also enable OPV to interact electrostatically with the negatively charged components present on the bacterial surface. Upon incubation with bacteria, OPV integrates tightly into the bacterial membrane through a combination of electrostatic and hydrophobic interactions. Experimental results demonstrate that OPV can rapidly generate ROS via both Type I (producing reactive oxygen free radicals such as H_2_O_2_ and (·OH) and Type II (generating ^1^O_2_) pathways under visible light irradiation, with the light-induced ROS generation disrupting membrane integrity and causing cell lysis. Cytotoxicity assays (MTT) indicate that OPV exhibits negligible toxicity toward MCF-7 cells under dark conditions (≤5 μM/L), demonstrating good biocompatibility. This study presents a novel strategy for developing efficient dual-mode photosensitizers effective in hypoxic environments [29].

#### 4.1.2. Photothermal Therapy

**Figure 4:**
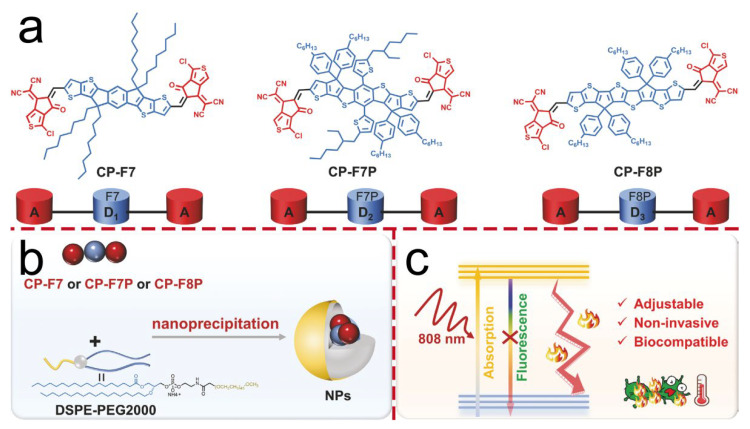
(**a**) Schematic diagram of the chemical structures of COs with three different donors. (**b**) Schematic diagram of the preparation of nanoparticles. (**c**) Mechanism diagram for photothermal antibacterial treatment. Heat is generated under the irradiation of an 808 nm laser. It has good adjustability, non-invasiveness, and biocompatibility.

The core mechanism of PTT involves the conversion of light energy into thermal energy, leading to the physical eradication of bacteria. In contrast to PDT, the excited-state energy is not released through the production of chemically active species, but rather through molecular vibrations and collisions that directly transform light energy into heat. When numerous photothermal agent molecules undergo this process simultaneously on or within bacterial cells, heat accumulates rapidly without sufficient time for dissipation, resulting in transient local hyperthermia. This intense thermal effect inflicts multi-target physical damage on bacterial cells: it disrupts the integrity of cell membranes, causing structural failure and leakage of intracellular components; it induces denaturation and coagulation of cytoplasmic proteins, resulting in enzyme inactivation and metabolic arrest; and it damages genetic material such as DNA. Collectively, these effects lead to rapid bacterial cell death [49,50,51]. The efficacy of PTT is predominantly determined by the photothermal conversion efficiency of the materials employed [52,53]. In the realm of conjugated materials, organic chromophores COs composed of donor and acceptor moieties play a pivotal role. Their photophysical properties, which are crucial for photothermal conversion, can be modulated by altering their molecular framework. This structural modification offers a strategic avenue for enhancing the photothermal performance of these materials, with significant implications for optimizing PTT outcomes. Yuan et al. explored the impact of the electron-donating capacity of the donor structure within molecular frameworks on photothermal conversion efficiency. They designed and synthesized three acceptor–donor–acceptor (A-D-A) type organic COs featuring identical acceptor units but varying donor structures within the main chain (Figure 4). By modulating the electronic properties of the donor or acceptor moieties, stronger electron-donating or electron-withdrawing characteristics were imparted to the molecules. Furthermore, enhanced intramolecular charge transfer was achieved, leading to improved absorption in the near-infrared (NIR) region and increased non-radiative heat generation. Among these, the CP-F8P donor, which possesses the highest degree of conjugation owing to the largest number of conjugated rings, exhibited the most pronounced electron-donating capacity and the highest photothermal conversion efficiency compared to the other two oligomers. Their research revealed that CP-F8P NPs, with robust electron-donating components, achieve a high photothermal conversion efficiency of 81.6% under an 808 nm laser. Experimental evidence indicates that at a concentration of 14 μg/mL, these NPs can elevate the solution temperature to 47.8 °C within 5 min of near-infrared irradiation. Remarkably, their bactericidal efficiency against Ampr *E. coli*, *S. aureus*, *C. albicans* and MRSA surpasses 99%. The antibacterial mechanism entails bacterial membrane disruption and nucleic acid leakage induced by the photothermal effect. In a diabetic mouse model with infected wounds, the combination of CP-F8P NPs and light irradiation markedly accelerated wound healing while exhibiting favorable biocompatibility. This investigation offers novel insights for designing near-infrared-activated antibacterial agents that are highly efficient and pose a low risk of drug resistance [19]. He et al. developed a quaternary ammonium salt-modified CO photosensitizer, RT-MN, for efficient photothermal treatment of bacterial infections. Conventional PTT often induces significant heating at the infection site; however, it typically lacks selectivity for bacterial targeting. Cationic electrostatic interactions offer a promising strategy for selective bacterial binding, owing to their ability to engage with negatively charged bacterial membranes. In this study, the authors designed and synthesized an organic molecule designated as RT-MN, functionalized with four cationic quaternary ammonium groups. This molecular design enables effective binding to bacteria through electrostatic interactions, thereby enhancing targeting specificity. Under 808 nm near-infrared laser irradiation, RT-MN generates heat, selectively eradicates bacteria, reduces inflammation in infected wounds, and promotes healing. Experimental data demonstrate that exposing bacteria to 150 μM RT-MN and an 808 nm laser results in nearly undetectable bacterial colonies, indicating effective inhibition of MRSA and *E. coli* growth. This study offers promising prospects for the clinical application of next-generation antibacterial agents [30].

#### 4.1.3. Synergistic Therapy

Nanocarrier technology has achieved significant breakthroughs in optical therapy, particularly in enhancing the synergistic effect of PTT and PDT. The synergistic antibacterial strategy combining PDT and PTT exhibits a superadditive effect—often conceptualized as “1 + 1 > 2”—stemming from the mutual enhancement of multiple physicochemical mechanisms. On one hand, the localized hyperthermia induced by PTT significantly increases the permeability of bacterial cell membranes. This not only causes direct physical disruption but also promotes the rapid penetration and diffusion of ROS generated via PDT into the bacterial interior, substantially enhancing their oxidative bactericidal efficacy. On the other hand, the thermal effect accelerates bacterial metabolic activity, rendering the cells more vulnerable to ROS-induced damage. Furthermore, the synergistic interplay allows for the use of reduced doses of photosensitizers and lower light intensities, thereby mitigating potential side effects such as tissue damage. This positions PDT/PTT combinatory therapy as a promising platform for the safe and effective treatment of refractory bacterial infections [54,55]. Lian et al. developed nanomaterial of a phthalocyanine-porphyrin oligomer (SiPc-ddCPP) based on orthogonal covalent linkage, which achieves highly efficient antibacterial effects through NIR photothermal-photodynamic synergistic therapy. This material is formed by amide bonds between the axial amino group of silicon phthalocyanine (SiPc-NH_2_) and the peripheral carboxyl group of porphyrin (ddCPP), creating a stable orthogonal structure. This structure overcomes traditional H-aggregation-induced blue shift and fluorescence/ROS quenching. Due to the inhibition of π-π aggregation, SiPc-ddCPP exhibits excellent solubility in both organic solvents and aqueous solvents. Experiments show that SiPc-ddCPP has a 31.15% photothermal conversion efficiency at 750 nm while retaining fluorescence emission and ROS generation (the ^1^O_2_ yield Φ = 0.17). In antibacterial experiments, under 660 nm (PDT) and 808 nm (PTT) irradiation, it completely kills Gram-positive (*S. aureus*) and Gram-negative (*E. coli*) bacteria within 5 min, with low toxicity to normal cells (MC3T3) (the survival rate is >85% at a concentration of 10 μM). Its antibacterial mechanism combines photothermal-induced membrane disruption and ROS-generated oxidative damage. Intramolecular energy transfer enhances NIR absorption and stability, offering a novel non-antibiotic-dependent strategy to overcome drug resistance [31].

This section systematically reviews the role of COs as an emerging class of antibacterial agents, which mediate efficient bacterial eradication through PDT, PTT, and synergistic therapeutic mechanisms. In contrast to conventional antibiotics and certain inorganic materials, the key advantages of COs lie in their multi-mechanism synergy, precise tunability of optoelectronic and structural properties, and markedly low potential for inducing drug resistance. The antibacterial action of COs operates not through single-target inhibition, but rather via a non-specific, multi-targeted physicochemical assault—combining oxidative damage, thermal ablation, and physical membrane disruption. This multi-faceted mode of attack greatly impedes the ability of bacteria to develop resistance through single-point mutations, thereby offering a robust strategy against drug-resistant pathogens.

### 4.2. Anticancer

As an emerging class of organic materials with tunable structures and exceptional optoelectronic properties, COs demonstrate considerable potential in the field of anticancer therapy. These materials mediate tumor ablation primarily through PTT and PDT mechanisms. Moreover, their capacity for functional modification allows them to function as targeted drug delivery vehicles, enabling precise transport and controlled release of chemotherapeutic agents. Certain COs can additionally elicit immune responses, further augmenting their antitumor efficacy. In summary, through a multimodal synergistic treatment strategy, COs enable a theranostic approach that integrates diagnostic and therapeutic functions, offering a promising direction for highly efficient and low-toxicity cancer treatment.

#### 4.2.1. Photothermal Therapy

Currently, the primary factor limiting the application of organic material PTT is their relatively low photothermal conversion efficiency, which stems from incomplete non-radiative transitions. The emergence of the acceptor–donor–acceptor structural motif has addressed this issue by not only enhancing light-harvesting capability but also promoting intramolecular charge transfer. This leads to fluorescence quenching and facilitates non-radiative decay, thereby significantly improving heat generation. Li et al. designed and synthesized a π-coupled oligomer-based nanoparticle (F8-PEG NP). Upon irradiation with an 808 nm laser, F8-PEG NP exhibited a marked temperature increase, achieving a photothermal conversion efficiency as high as 82%. Moreover, the absorption profile of F8-PEG NP remained largely unchanged after photothermal treatment, confirming excellent photostability. In vitro experiments demonstrated that F8-PEG NP not only induced highly efficient ablation across three cancer cell lines (A549, 4T1, and HeLa), but also accomplished complete tumor eradication in mouse models, underscoring its superior phototherapeutic efficacy. Importantly, F8-PEG NP can be biodegraded by endogenous enzymes, effectively addressing long-term toxicity concerns associated with synthetic materials. In conclusion, in this study we not only introduce a highly efficient photothermal agent but also provides valuable insights for the rational design of biodegradable materials aimed at effective cancer therapy [32].

#### 4.2.2. Combination Therapy

**Figure 5:**
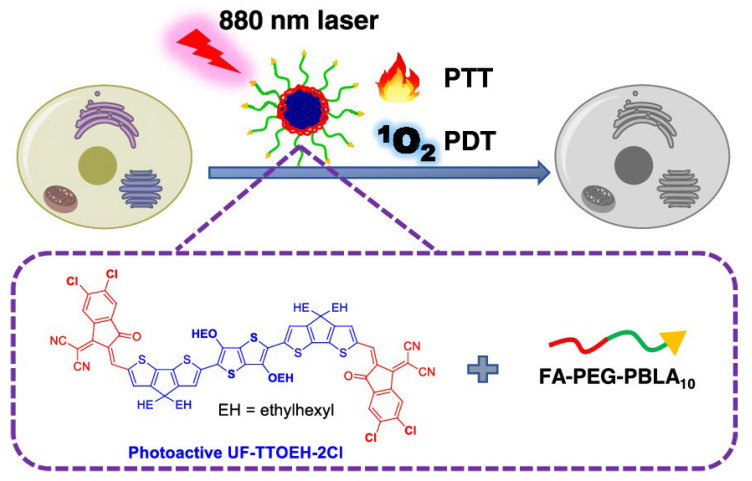
Schematic diagram of the mechanism of synergistic PTT and PDT for tumors under the irradiation of an 880 nm laser. Molecular structure diagram of the dual-functional phototherapy oligomer UF-TTOEH-2Cl optimized based on CPDT [12]. Reprinted with permission from [12]. Copyright 2023 American Chemical Society.

COs featuring an A-D-A architecture play a critical role in enhancing PTT/PDT combination therapy for antitumor applications, with their core advantage stemming from precise performance optimization through rational molecular design. This structural motif effectively reduces the energy gap of the molecule via the alternating arrangement of strong electron-donating (D) and electron-accepting (A) units, enabling absorption tuning into the near-infrared region. As a result, deeper tissue penetration and minimized photodamage to normal tissues are achieved. More importantly, the A-D-A framework significantly promotes intramolecular charge transfer (ICT), which not only enhances light-harvesting efficiency, but also concurrently optimizes two key therapeutic properties: the production volume of singlet oxygen and AIE. Zhao et al. developed a photoactive A-D-A-type CO UF-TTOEH-2Cl, and prepared a near-infrared (NIR)-responsive nanoparticle platform UTNPs (Figure 5). It showed high photothermal conversion efficiency (42%) and singlet oxygen (^1^O_2_) yield. Constructed on the original photoactive material CPDT, this material inserted thieno [3,2-b]thiophene (TT) between the two DTC cores, extended the conjugated main chain, and added four chlorine atoms to INCN for bandgap regulation; a PTT/PDT-functional phototherapeutic oligomer was synthesized. Compared to CPDT, UF-TTOEH-2Cl exhibits a pronounced bathochromic shift in absorption, with a peak maximum λ_max_ at 825 nm within the 600–950 nm region. Correspondingly, it possesses a narrower optical band gap of 1.82 eV relative to that of CPDT (1.91 eV). These characteristics facilitate enhanced utilization of NIR light. The photothermal conversion efficiency (PTCE) of UTNP was determined to be about 42% via heating and cooling cycles. The electron spin resonance technique (ESR) was used to determine that the type of ROS generated was ^1^O_2_. Here, we have not only designed a dual-functional light-responsive material for NIR light-triggered PDT/PTT synergistic tumor therapy, but also revealed the influence of the A-D-A skeleton on the ROS generation and photothermal properties of photosensitive materials [12].

In PDT, ROS play a crucial role in eradicating bacteria and tumor cells. However, excessive ROS can damage biological macromolecules and trigger cell necrosis. To counteract this, the cellular antioxidant system is activated to alleviate oxidative stress and prevent ROS accumulation. Thus, developing materials to modulate signaling pathways, diminish the cell’s antioxidant capacity, and boost PDT efficacy is highly significant [56,57]. The Keap1-Nrf2 signaling pathway is pivotal in regulating cellular oxidation and xenobiotic metabolism, particularly in tumor cells where it enhances antioxidant capacity [58]. Creating chemical materials that can modulate this pathway and increase oxidative stress is crucial for improving anti-tumor outcomes.

**Figure 6:**
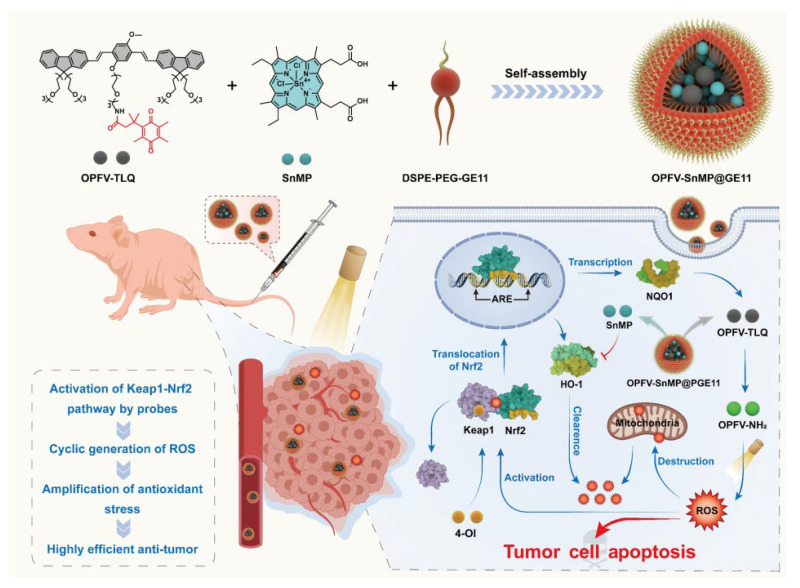
Schematic diagram of the preparation of the conjugated oligomer nanoparticle OPFV-SnMP@GE11. Schematic diagram of the regulation mechanism of the nanoparticle on the Keap1-Nrf2 pathway, which is used to cyclically enhance the killing ability of tumor cells [33]. Copyright and Permissions 2023 Chinese Chemical Society.

Bao et al. presented a nanostrategy to enhance oxidative stress by modulating the Keap1-Nrf2 antioxidant pathway for anti-tumor purposes. They constructed a multifunctional nanoparticle OPFV-SnMP@GE11 (Figure 6). These nanoparticles accumulate at the tumor site via GE11-mediated EGFR-targeting. Tumor cells’ highly expressed quinone oxidoreductase 1 (NQO1) specifically reduces OPFV-TLQ to the photosensitizer OPFV-NH_2_. Upon light irradiation, OPFV-NH_2_ generates ROS through a type I photodynamic mechanism. This ROS activates the Keap1-Nrf2 pathway, promoting NQO1 transcription and forming a ROS-NQO1 positive feedback loop for an explosive ROS increase. In vitro, this strategy significantly reduces mitochondrial membrane potential and induces apoptosis (the apoptosis rate is 97%). Western blotting shows that the expressions of the Nrf2, NQO1, and p53 proteins are significantly upregulated. In vivo, the nanoparticles effectively inhibit tumor growth under light irradiation, with notable tumor volume reduction and good biosafety. This study offers novel ideas for anti-tumor treatment by enhancing oxidative stress through signaling pathway regulation [33].

Chemotherapy, the most common clinical treatment for tumors, lacks specificity for tumor cells [59,60]. This can cause systemic toxicity and lead to drug resistance. Therefore, it is crucial to achieve precise treatment by accurately controlling drug release in specific areas and at specific times [61]. Wang et al. designed a strategy to overcome chemotherapeutic drug efflux via the intracellular assembly and aggregation of COE. They synthesized a cationic conjugated oligoelectrolyte OPV-S-PTX by modifying a hydrophobic oligo (phenylene vinylene) backbone (OPV-SH) with a thiol group and the anticancer drug paclitaxel. The π-π stacking between main units facilitates aggregation in aqueous solutions. PTX can promote the polymerization of tubulin, alter normal cell functions, induce abnormal mitosis of cells, and ultimately lead to the apoptosis of tumor cells. The thiol groups, responsive to oxidative stress, enable disulfide bond cross-linking to form stable large aggregates. In cancer cells with high ROS levels, OPV-SH forms aggregates. ROS oxidizes the thiol groups, triggering intermolecular cross-linking within the aggregates. This confines OPV-SH within the cells for a sustained anticancer effect. Modifying OPV-SH with paclitaxel enhances toxicity to tumor cells. Notably, OPV-S-PTX is assembled within cells and then cross-linked through thiol functional groups in the presence of ROS to generate nanoparticles. This cross-linking process occurs in the cytoplasm of cancer cells but not in healthy mammalian cells, and it provides a basis for inhibiting the diffusion of drugs from cells and enhancing therapeutic effects. This study offers a novel strategy to combat drug resistance, lower drug toxicity, and decrease drug diffusion out of cells [34].

This section examines the application of COs in antitumor therapy, highlighting their considerable promise as a novel class of organic materials with tunable structures and outstanding optoelectronic properties. The antitumor mechanism of COs primarily involves PTT and PDT, which are efficiently synergized through rational molecular design. Beyond combined PTT/PDT, the therapeutic mechanisms of COs also extend to modulation of the tumor microenvironment and activation of specific signaling pathways. These features substantially enhance the efficacy of PDT and underscore the potential of COs for precise biomedical regulation.

## 5. Conclusions

COs offer several advantages in the medical field, including the following: (1) Structural versatility and functional diversity. By precisely regulating the conjugation length of the main chain and the types of side-chain groups, their light absorption capacity, targeting ability, antimicrobial properties, and catalytic activity can be tailored. This enables better applications in deep-tissue imaging and therapy. (2) Excellent optoelectronic properties and stability. They exhibit higher fluorescence quantum yields and stronger resistance to photobleaching, allowing for long-term, real-time monitoring of living cells. (3) Superior biocompatibility and metabolic efficiency: The small-molecule nature of COs enables rapid renal excretion, reducing long-term biological toxicity.

However, the clinical translation of COs continues to face several systemic challenges. The primary concern lies in the insufficient understanding of their in vivo behavior and long-term biosafety, particularly the potential risk of chronic accumulation due to unclear biodistribution, metabolic pathways, and non-biodegradable nature. Secondly, at the material level, the reproducible batch synthesis of structurally complex COs remains difficult to achieve. Moreover, balancing hydrophilic modification with optimal optoelectronic properties poses a persistent design challenge. From an application perspective, the efficacy of phototherapy is constrained by the limited depth of tissue that light can penetrate. Furthermore, the hypoxic tumor microenvironment can significantly compromise PDT outcomes, and existing targeting strategies still suffer from off-target risks.

In response to these challenges, future research efforts should focus on several strategic directions. Innovative molecular design incorporating biodegradable linkages offers a promising avenue to address long-term safety concerns. The development of smart theranostic platforms responsive to the tumor microenvironment could enable precise activation and enhance specificity. To overcome depth limitations, exploring alternative activation modes using external stimuli such as X-rays or ultrasound represents an emerging trend. Additionally, constructing dual-mode COs that integrate diagnostic and phototherapeutic functions through modular design may pave the way for all-in-one diagnosis-treatment-monitoring systems. Such advances are expected to transform the landscape of cancer therapy and significantly broaden the application prospects of COs in healthcare.

## Figures and Tables

**Table 1:** Summary of COs for imaging, antibacterial and antitumor.

Material	Laser	Type	In Vivo	In Vitro	Ref.
OT-Se	White light, 30 mW/cm^2^	PDT in hypoxic environments	Trauma surface injection, BALB/c mice	*CREC* */* *MRSA*	[11]
UF-TTOEH-2Cl	NIR (880 nm), 0.8 W/cm^2^	PDT/PTT combination therapy	-	HeLa cells/SiHa cells	[12]
CP-F8P NPs	808 nm, 1 W/cm^2^	Kill bacteria by PTT	Trauma surface injection, diabetic mice with wounds	*E. coli/S. aureus/C. albicans/MRSA*	[19]
COE-BBT	NIR-II region (1000–1700 nm)	Fluorescence detection	An orthotopic glioma model, BALB/c mice	A549 cells/C6-Luc glioma cells	[20]
Supramolecular Fluorescent Sensor Array	431 nm	Fluorescence detection	-	10 distinct bacterial strains	[25]
COE-S6	405 nm	Fluorescence detection	-	*E. faecalis* OG1RF/*E. coli* UTI89	[26]
O-T, O-DT and O-Q	1064 nm, 1 W/cm^2^	NIR-II fluorescence detection	Xenograft, tumor-bearing mice	Hela cells	[27]
N4 NPs	600–900 nm (808 nm, 0.8 W/cm^2^)	Photoacoustic detection	Injection of the left forepaw pad, Wistar rat	MDA-MB-231 breast cancer cells/NIH-3T3 mice cells	[28]
OPV	422 nm, 90 mW/cm^2^	PDT	-	*E. coli/MRSA*	[29]
RT-MN	808 nm, 1 W/cm^2^	Highly efficient PTT of bacterial infections	Artificial trauma and injection of bacteria, BALB/c mice	*E. coli/MRSA*	[30]
SiPc-ddCPP	NIR (808 nm), 3.5 W/cm^2^	PDT/PTT combination therapy	-	*S. Aureus/E. coli*	[31]
F8-PEG NPs	NIR (808 nm), 1 W/cm^2^	PTT	4T1 tumor xenograft	A549/4T1/HeLa cells	[32]
OPFV-SnMP@GE11	418 nm, 25 mW/cm^2^	PDT/signaling pathway modulation	Subcutaneous cell injection	A431 cells	[33]
OPV-S-PTX	418 nm	Synergistic PDT-chemotherapy therapy	Xenograft, BALB/c mice	A549/T cells and MCF-7m cells	[34]

## Data Availability

Not applicable.
